# Empagliflozin-induced Diabetic Ketoacidosis Unmasking a Type 1 Diabetes Diagnosis

**DOI:** 10.5811/cpcem.2019.2.41795

**Published:** 2019-03-05

**Authors:** Gretchen M. Ray, Chelsea Rodriguez, Samantha M. Schulman, Preeyaporn Sarangarm, Michelle Bardack, Matthew F. Bouchonville

**Affiliations:** * The University of New Mexico College of Pharmacy, Department of Pharmacy Practice and Administrative Sciences, Albuquerque, New Mexico; † The University of New Mexico College of Pharmacy, Albuquerque, New Mexico; ‡ The University of New Mexico Hospitals, Department of Pharmacy, Albuquerque, New Mexico; § The University of New Mexico School of Medicine, Department of Family and Community Medicine, Albuquerque, New Mexico; ¶ The University of New Mexico School of Medicine, Department of Internal Medicine, Division of Endocrinology, Diabetes, and Metabolism, Albuquerque, New Mexico

## Abstract

Empagliflozin is a sodium glucose cotransporter-2 inhibitor that inhibits renal glucose reabsorption through an insulin-independent mechanism. This class of drugs is used in the management of type 2 diabetes. A 49-year-old female with type 2 diabetes treated with empagliflozin presented to the emergency department in diabetic ketoacidosis (DKA). This case report details the series of events leading to the diagnosis of drug-induced DKA, which led to a change in the patient’s diagnosis from type 2 diabetes to type 1 diabetes.

## INTRODUCTION

The treatment of diabetes has rapidly evolved with the introduction of novel agents such as the sodium glucose cotransporter-2 (SGLT2) inhibitors. In 2013, the United States (U.S.) Food and Drug Administration (FDA) approved the first SGLT2 inhibitor, canagliflozin, for the treatment of non-insulin-dependent type 2 diabetes mellitus (T2DM). Since that time, three additional agents, empagliflozin, dapagliflozin, and ertugliflozin, have begun to be marketed in the U.S. These agents are now included into the American Diabetes Association (ADA) treatment guidelines as one of six possible add-on pharmacologic agents to metformin.^1^ SGLT2 inhibitors are effective for the treatment of T2DM as they inhibit renal glucose reabsorption through an insulin-independent mechanism, which in turn lowers glucose levels through increased urinary glucose excretion.^2^ This drug class is also associated with a reduction in body weight, as well as reduced blood pressure, which is largely due to their natriuretic effect.^2^ Additionally, two SGLT2 inhibitors, empagliflozin and canagliflozin, have been shown to reduce rates of major adverse cardiac events in high cardiovascular-risk patients in the Empagliflozin, Cardiovascular Outcomes, and Mortality in Type 2 Diabetes trial and Canagliflozin and Cardiovascular and Renal Events in Type 2 Diabetes (CANVAS) trials.^3^,^4^ Accordingly, prescriptions for SGLT2 inhibitors have been on the rise, resulting in this class of medications being commonly encountered in the primary care and emergency department (ED) settings.^5^

Common side effects of this drug class include increased risk of urinary tract infections, genital mycotic infections, and volume depletion.^1^ Both diabetic ketoacidosis (DKA) and euglycemic diabetic ketoacidosis (euDKA) have since also been identified as rare but serious adverse effects of the SGLT2 inhibitors, and in 2015 the FDA released a safety alert to the public about this concern.^6^ In contrast to the low rates of DKA observed in SGLT2 inhibitor users with type 2 diabetes, the risks are remarkably higher in those with type 1 diabetes.^7^ This distinction has prompted a consensus statement by the American Association of Clinical Endocrinologists urging caution with the off-label use of SGLT2 inhibitors in patients with type 1 diabetes.^8^ We describe the case of a patient initially diagnosed with type 2 diabetes presenting in DKA in association with the use of an SGLT2 inhibitor.

## CASE REPORT

A 49-year-old female presented to the ED after waking up with nausea and abdominal pain followed by multiple episodes of vomiting. Her past medical history included T2DM, diagnosed four years earlier, and hypertension. Antihyperglycemic medications at the time of presentation included insulin glargine 25 units subcutaneous once a day, exenatide 10 micrograms (mcg) subcutaneous twice a day, empagliflozin 25 milligrams (mg) once a day (started four months prior to admission), and metformin 1000 mg twice a day.

Pertinent laboratory values upon presentation to the ED included the following: hemoglobin A1C 10.5% (4.4–5.6%), glucose 251 mg/deciliter (dL) (60–100 mg/dL), chloride 93 millimols per liter (mmol/L) (98–111 mmol/L), carbon dioxide 12 mmol/L (20–30 mmol/L), anion gap 29 (6–14), c-peptide 0.1 nanogram per milliliter (ng/mL) (0.9–6.9 ng/mL), ketone beta-hydroxybutyrate > 2.0 mmol/L (0.02–0.27 mmol/L), serum osmolality 322 milliosmoles per kilogram (mOsmol/kg) (280–295 mOsmol/kg), lactate 2.7 mmol/L (0.4–2.0 mmol/L) and a urine analysis with abnormal glucose of 500 mg/dL and ketones 80 mg/dL, but otherwise unremarkable. She was diagnosed with DKA and admitted to the intensive care unit on intravenous hydration and insulin drip per institution protocol.

DKA resolved two days following admission and the patient was discharged. At discharge, no precipitating factor leading to her DKA had been identified during the hospitalization. There had been no evidence of infection or pancreatitis, and she was discharged on all home medications with an increase in her insulin glargine to 30 units once a day.

She was seen in her primary care clinic six days post-discharge. Additional laboratory values were drawn including glutamic acid decarboxylase (GAD) antibody, which was elevated > 250 units/mL (< 0.5 units/mL). Given that the empagliflozin had been initiated four months prior to her hospital admission and that she had been admitted with euDKA with a glucose level of only 251 mg/dL at presentation, at the primary care follow-up, it was determined that this was a case of SGLT2 inhibitor-induced DKA. She had been managed as a type 2 diabetic for four years, but her low c-peptide level and elevated GAD antibody drawn at this post-discharge follow-up appointment resulted in a change in diagnosis to type 1 diabetes from type 2. All non-insulin antihyperglycemic agents including the empagliflozin that precipitated the DKA were discontinued and she was placed on a basal plus bolus insulin regimen.

**CPC-EM Capsule**

What do we already know about this clinical entity?

*Sodium glucose cotransporter-2 (SGLT2) inhibitors are commonly used for the management of type 2 diabetes. This drug class has a known, rare side effect of diabetic ketoacidosis (DKA)*.

What makes this presentation of disease reportable?

*The SGLT2 inhibitor-induced DKA led to the unmasking of type 1 diabetes in a patient previously diagnosed with type 2 diabetes*.

What is the major learning point?

*DKA is a rare risk of the SGLT2 inhibitor drug class, and there are certain factors that may predispose the patient to this adverse event. In this case, the underlying risk was type 1 diabetes*.

How might this improve emergency medicine practice?

*Improve identification of potential drug-induced DKA and underlying risk factors that may predispose the patient to this adverse effect*.

## DISCUSSION

The ADA diagnostic criteria for DKA include hyperglycemia (blood glucose > 250 mg/dL), metabolic acidosis (arterial pH < 7.3 and serum bicarbonate < 18 mEq/L) and ketosis.^9^ Euglycemic DKA was originally described in the literature as severe ketoacidosis with a blood glucose level less than 300 mg/dL, but currently a more common definition is a blood glucose < 200 mg/dL.^10^,^11^ SGLT2 inhibitors have been hypothesized to lead to this condition as a result of glucosuria leading to a rapid reduction in plasma glucose levels.^12^,^13^ This reduction in plasma glucose leads to decreased insulin release from the beta cells, which then leads to stimulation of alpha cells and the increase in plasma glucagon concentrations further stimulating hepatic ketogenesis.^12^,^13^ These mechanisms of ketogenesis in combination with continued glucosuria lowering plasma glucose levels results in the presence of ketone bodies in the setting of normal glucose levels.^13^ A total of 73 cases of SGLT2 inhibitor-related ketoacidosis were identified during a review of the FDA Adverse Event Reporting System database from March 2013 to May 2015. All patients required hospitalization or treatment in the ED, and many cases were complicated by a delayed diagnosis due to the low blood glucose levels on presentation.^6^

The SGLT2 inhibitors have also been studied for use in patients with type 1 diabetes.^14^–^16^ Their insulin-independent mechanisms offer an attractive and likely effective option as an add-on to insulin therapy. It has been hypothesized that patients with autoimmune type diabetes (latent autoimmune diabetes of adulthood, or type 1 diabetes) would be at greater risk of DKA in the setting of SGLT2 inhibitor therapy given their lack of endogenous insulin production leading to inability to overcome the SGLT2 inhibitor-induced increase in glucagon, thus leading to the setting of unsuppressed hepatic ketogenesis.^17^ A recent systematic review aimed to identify precipitating factors of SGLT2 inhibitor-induced DKA.^13^ In this review, two-thirds of all cases involved patients with T2DM; however, nine out of 25 of those individuals were later diagnosed with latent autoimmune diabetes of adulthood following resolution of their DKA.^13^ Additionally, an analysis of several cases of DKA in patients who were taking canagliflozin for type 2 diabetes in the CANVAS trial series found that six out of the 12 patients were diagnosed with autoimmune diabetes or tested positive for GAD65 antibodies after the development of DKA.^17^

Our case also describes a patient with presumed type 2 diabetes who, following her resolution of SGLT2 inhibitor-induced DKA, was further evaluated with antibody testing, and was revealed to have type 1 diabetes based on the presence of GAD antibodies. She had been treated as a type 2 diabetes patient for four years prior to her episode of DKA. Additionally, the causative agent, or precipitating factor, had not been identified at initial presentation to the hospital, or during the hospitalization. Although medications are not often identified as the precipitating factor for DKA, the SGLT2 inhibitors are being implicated with increased frequency in cases of DKA. In this case, the patient was discharged on the empagliflozin placing her at risk for a repeat event. The changes in diagnosis from type 2 to type 1 diabetes occurred after discharge at the primary care office.

## CONCLUSION

We report a case of DKA secondary to the use of empagliflozin that resulted in a change in diagnosis from type 2 diabetes to type 1 diabetes. This case also highlights a situation in which the diagnosis of SGLT2 inhibitor-induced DKA was not made while the patient was in the hospital and she was discharged on the offending agent, thus placing her at risk for a repeat event. Such an event indicates the need for emergency and critical care providers to remain vigilant in identifying drug-induced causes of DKA.
